# Calcined Post-Production Waste as Materials Suitable for the Hydrothermal Synthesis of Zeolites

**DOI:** 10.3390/ma12172742

**Published:** 2019-08-27

**Authors:** Michał Łach, Agnieszka Grela, Norbert Komar, Janusz Mikuła, Marek Hebda

**Affiliations:** 1 Institute of Materials Engineering, Cracow University of Technology, Warszawska 24, 31-155 Kraków, Poland; 2 Department of Water Engineering and Management, Cracow University of Technology, Warszawska 24, 31-155 Kraków, Poland; 3 Ekologia Przedsiębiorczość Innowacje Spółka z o.o., 42-256 Olsztyn, Poland

**Keywords:** zeolite synthesis, coal shale, metakaolin, thermal analysis, calcination, water leaching

## Abstract

The zeolite production process is currently being very intensively researched. Due to environmental protection, as well as issues related to the guidelines of a zero-waste economy, all activities aimed at obtaining such materials from post-processed waste are extremely important. This article presents an innovative method of utilising calcined carboniferous shale in order to produce synthetic zeolites. The raw material for testing came from two Polish hard coal mines. Both the chemical and phase composition of the coal shale were characterised. Based on the recorded thermal analysis results coupled with the mass spectrometer, the processes occurring during the heating of raw materials were interpreted and the calcination temperatures were determined. The changes in the phase composition of raw materials resulting from the calcination process used were also analysed. The heat-treated raw materials were subjected to the synthesis of zeolites in an aqueous solution of sodium hydroxide by means of the hydrothermal method at a concentration of 2.75 M. The results of water leaching and structural parameters are presented for both raw materials, as well as the produced synthesis. The conducted research confirmed that after the application of the synthetic process on coal shale, a zeolite with a surface area of *S_BET_* equal to 172 m^2^/g can be obtained.

## 1. Introduction

The intensive development of the industry, as well as the need to satisfy constantly growing human needs, have resulted in the generation of significant amounts of waste. Thus far, in the case of mining waste, the most popular method of its management has been in depositing it in waste dumps or placing it in mining excavation sites. However, increasing attempts are being made to process such waste, which is conducive not only to environmental protection but also to the possibility of recycling secondary raw material [1]. Approximately 20 million tonnes of waste is generated annually in Poland. Across the whole of Europe, this amounts to 700 million tonnes, of which approximately 150 million tonnes are by-products of coal burning in the energy sector. The use and reprocessing of waste are in line with the regulation: Zero Waste Europe, Resource Efficient Europe, and Circular Economy [2].

Such an energy raw material as hard coal will remain as one of the most important energy carriers, enabling the development of the economy over the next few decades [3]. This applies to both the global as well as regional scale, as described, for example, during the last World Energy Outlook 2017 of the International Energy Agency. In connection with the above, large quantities of post-production waste, as well as by-products of combustion, will still be generated globally [4,5,6,7,8].

At present, the management of coal shale (waste rocks) is carried out by burning it for energy recovery or by utilising it in construction, among other things, as an admixture for cement. Combustion of coal shale contributes to the utilisation of its energy potential as it can be considered as a substitute fuel. In addition, it can also be utilised as a substitute for the raw material used in the clinker burning process. Despite the aforementioned possibilities of using coal shale, new and more rational methods and technologies of its management are continually being looked for [9,10]. Due to the poor pozzolanic activity of shale rock, it is necessary to subject it to the thermal processing of calcination [11,12,13,14,15]. During the calcination process of waste coal shale, dehydroxylation of clay minerals, such as kaolin, occurs. This leads to the formation of metakaolin, which is an amorphous phase with high reactivity (pozzolanic activity) [13,16,17,18]. The main components of coal shale are kaolinite, quartz, illite and unburned coal. Analyses conducted by using the coupled thermogravimetric with differential scanning calorimetry (TG-DSC) method showed that kaolinite was converted to an amorphous phase at a temperature of 500 °C [19]. In the temperature range of 600–800 °C, the plaque structure of kaolinite is degraded and forms into an irregular and porous phase; it then crystallises into mullite when the temperature reaches over 1000 °C [13,19]. Pozzolanic reactivity can also be improved by chemical activation, e.g., by introducing CaSO_4_ and CaO [20].

Thanks to the high content of clay minerals, as well as the possibility of their thermal transformation, coal shale has a high reactivity and can be an attractive material for the production of sorbent materials. The production of sorbent materials, such as zeolites, is an innovative way of managing this type of waste.

Zeolites are crystalline, hydrated alkaline earth metal aluminosilicates. They are divided into: (i) natural, created by the weathering of volcanic rocks, which currently comprise approximately 40 types; and (ii) synthetic. The interest in synthetic zeolites increases as more and more new possible applications of these minerals in various fields of industry are discovered [21]. 

The zeolite application in construction has been used for a long time, primarily due to their excellent mechanical properties [22]. Namely, their absorption of water, porosity, specific weight and mechanical properties are parameters that enhance the application of the zeolite in constructions. There are several studies that have taken place related to the mechanical properties of zeolites pertaining to their use in construction, as a foundation basis, or as masonry blocks in buildings [23,24]. Zeolites A are commonly used primarily to remove various contaminants in environmental technologies. According to literature sources, this material is referred to as a high-quality mineral sorbent used to remove ammonium ions [25,26] and heavy metals [27,28] from aqueous solutions. Other studies also focus on the use of type A zeolites for the adsorption of various types of gases, including NO_x_, SO_x_ and CO_2_ [29,30].

Advanced synthesis methods allow for the production of zeolitic materials with strictly defined parameters that are adapted to different applications. The synthesis methods include: low- and high-temperature hydrothermal synthesis, alkaline fusion or the molten salt method, as well as two-stage synthesis [31,32]. The research and development activities that have been carried out for decades are focused on searching for affordable and commonly available substrates, as well as for reducing the costs of the reaction itself. Currently, clay minerals and reactive silica are used as substrates in the synthesis of zeolites. Fly ash is also regarded as an attractive raw material due to its similarity to zeolites in terms of chemical composition and the presence of reactive phase components, mainly mullite [31,32]. The issue concerning the rational use of by-products from mining and coal combustion is currently being given a considerable amount of attention due to the environmental and social risks that this waste generates. Fly ash, as well as other post-production waste that is to be used in the synthesis of zeolites, are predisposed by a significant Si and Al content, allowing the production of an effective sorbent material with a limited spectrum of applications. 

The currently available literature only describes the possibilities of obtaining coal shale sorbents [33,34,35,36]. For example, Qian et al. describes the synthesis of a highly crystalline Na-A zeolite phase with a high Ca^2+^ ion exchange capacity after the use of thermal and hydrothermal activation [33]. Furthermore, Gao et al. developed a method to produce new porous silicon material (PSM) synthesised from coal shale [34]. 

Moreover, the process of synthesis of zeolites from the coal gangue has already been analysed by other authors; however, the research methodology presented in this article was never applied. For example, Chenet al. [37] presented a method of obtaining zeolites from coal gangue through the alkaline fusion method. Their results show that NaA zeolite was successfully synthesised by alkaline fusion at 650 and 950 °C, while NaX zeolite was synthesised by alkali melting at 600 °C. A similar method of synthesis of zeolites was used by Qian et al. [33]. They obtained pure single-phase and highly crystalline Na-A zeolite with high Ca^2+^ exchange capacity (CEC). The mixing process was carried out for 20 min and next heated at 400 °C for 2 h. Then, they conducted a hydrothermal synthesis, changing the temperature from 80 to 100 °C and the NaOH concentrations from 1.0 to 3.5 mol/L. Another method of obtaining zeolites from coal shales is the method described by Jinfeng et al. [38]. They used an ultrasonic treatment, where the frequency and power of ultrasound were 20 kHz and 120 W, respectively. Thanks to the combination of ultrasonic pretreatment, combined with hydrothermal growth method, zeolite SSZ-13 was synthesised.

The studies described in this paper show the effectiveness of hydrothermal synthesis methods for developing post-production waste by utilising hard coal shale to produce zeolites without the need for a fusion method (high-temperature method) or pre-treatment, e.g. using ultrasound. 

Furthermore, despite the fact that the methods for the synthesis of zeolites with coal gangue are known, they are still optimised in order to maximise economic efficiency. Only the development of an easy and cheap method of synthesis of zeolites from waste materials that differs in composition depending on the location of deposits, which in many countries are found in dumps near mines, may allow for their industrial processing into zeolites and thus for economic use.

## 2. Materials and Methods

Samples for testing were taken from two Polish hard coal mines: Piast (Bieruń, Silesian Voivodeship, Poland) and Ruch Rydułtowy (Rydułtowy, Silesian Voivodeship, Poland). The material was first crushed in a jaw crusher and then milled in a ZM200 RETSCH (Retsch, Hann, Germany) ultra centrifugal mill (0.040 mm sieve).

On the basis of the measurements of thermal analysis described in the text, it was found that the optimal temperature of the calcination process of the raw materials tested was 750 °C. The materials were kept at this temperature for 240 min.

Next, in the cylindrical vessels, made of polypropylene with a volume of 1 dm^3^, milled coal-bearing shale was mixed with a 2.75 M water solution of sodium hydroxide NaOH (purity > 98%). For every 30 g of material, 100 mL of a 2.75 M NaOH solution was used. A molar concentration equal to 2.75 M NaOH was chosen based on previous own experiments, as well as based on the results presented in the literature [39,40,41]. Loiola et al. [40] conducted a synthesis using a concentration of 2.75 M NaOH. Also, Gougazeh et al. obtained the best results in the synthesis of zeolite A using concentrations in the range between 2.5 and 3.0 M NaOH [41]. The synthesis process was carried out in tightly closed vessels at a temperature of 80 °C for 24 h. At the same time, the samples were filtered in order to remove the remaining solution.

After the filtration, the samples were washed using distilled water in order to achieve a pH of about 9. The filtration and washing were conducted with filter paper dedicated to qualitative analysis. Finally, the samples were dried at 105 °C for 6 h. Table 1 shows the description of the samples.

The morphology of the samples was examined by means of the scanning electron microscope JEOL JSM 820 (Tokyo, Japan). Samples were put on the coal base and covered with a thin layer of gold with the JEOL JEE-4X vacuum evaporator (Tokyo, Japan) to carry the electric charge.

XRD analysis was recorded with the use of a Rigaku SmartLab X-ray diffractometer (Tokyo, Japan) using the following parameters: CuKα radiation, a graphite reflection monochromator, tube voltage = 45 kV, tube current = 200 mA, step size = 0.05° 2θ, count time per 1 step = 1s. The values of interplanar distances obtained from XRD patterns were used for the identification of phases present in the samples based on data contained in the ICDD (International Centre for Diffraction Data 2014) Catalogue and XRAYAN software (v.4.0.1, Warsaw, Poland). 

Simultaneous thermogravimetric (TG) and differential thermal analysis (DTA) measurements were carried out using a Netzsch STA 449F3 Jupiter apparatus (Selb, Germany). Fifty milligrams of an air-dried sample was heated from 20 to 1000 °C in an alumina crucible at 10 °C min^−1^ in flowing (40 mL min^−1^) synthetic air. Analyses of the evolved gases were carried out using a Netzsch QMS 403C Aȅolos quadrupole mass spectrometer. The apparatus was calibrated in accordance with the methodology presented in References [42,43].

The chemical composition of the samples was determined by means of X-ray fluorescence (WD-XRF) on a ZSX Primus II Rigaku spectrometer (Tokyo, Japan). Spectral analysis was performed by identifying spectral lines and determining their possible coincidences. Quantitative analysis was formulated using the SQX Calculation program (fundamental parameters method) (Rigaku, Tokyo, Japan). The analysis was carried out in the fluorinated (FU) range and the contents of the elements were normalised to 100%. 

The ignition losses were determined by calcining the sample at 950 °C and calculating the difference in mass, both before and after calcining the sample.

Porosity and specific surface area measurements were performed with the use of a Brunauer–Emmett–Teller (BET)-accelerated surface area and porosimetry analyser ASAP 2020 (Micromeritics, Norcross, GA, USA). Samples were degassed at a temperature of 373 K for 24 h. To determine the distribution of pore volume function in the materials analysed, experimental low-temperature (77 K) nitrogen adsorption isotherms were used. The Barrett–Joyner–Halenda (BJH) method was used to determine the mesopore volume and to determine the specific surface area, the BET method was applied.

In order to determine the leachability of easily mobile elements, the samples were subjected to a water leaching test in accordance with the PN-EN 12457-4: 2006 standard. The weight of undried material with a total mass corresponding to 0.090 kg (dry weight) and grain size less than 10 mm was submerged in distilled water in a liquid/solid ratio of 10 dm^3^/kg and shaken for 24 h. After this time the samples were subjected to 15 min of sedimentation. It was then filtered through a 0.45 μm membrane filter using a pressure filter device. The analysis of the chemical composition of the solution was carried out by means of emission and mass spectrometry with inductively excited plasma (ICP-OES, ICP-MS) on a Perkin Elmer Plasm 40 (Waltham, MA, USA) and ELAN6100 spectrometers (Waltham, MA, USA). The content of SO_4_ sulphates, Cl chlorides and HCO_3_ hydrogen carbonates was determined using turbidimetry, argentometric titration and alkalimetric titration, respectively. 

## 3. Results and Discussion

Table 2 presents the chemical composition of the tested coal shale. They were characterised by the dominant content of SiO_2_ at about 66% for K1 and 57% for K2. The second component in terms of content was Al_2_O_3_, which occurred in the amount of 21.44% in sample K1 and 27.37% in K2. Measured contents of said compounds, as well as their mutual ratio, which were almost three times greater than SiO_2_ to Al_2_O_3_, indicated the potential usefulness of this type of materials as a raw material for the synthesis of zeolites [44].

**Figure 1:**
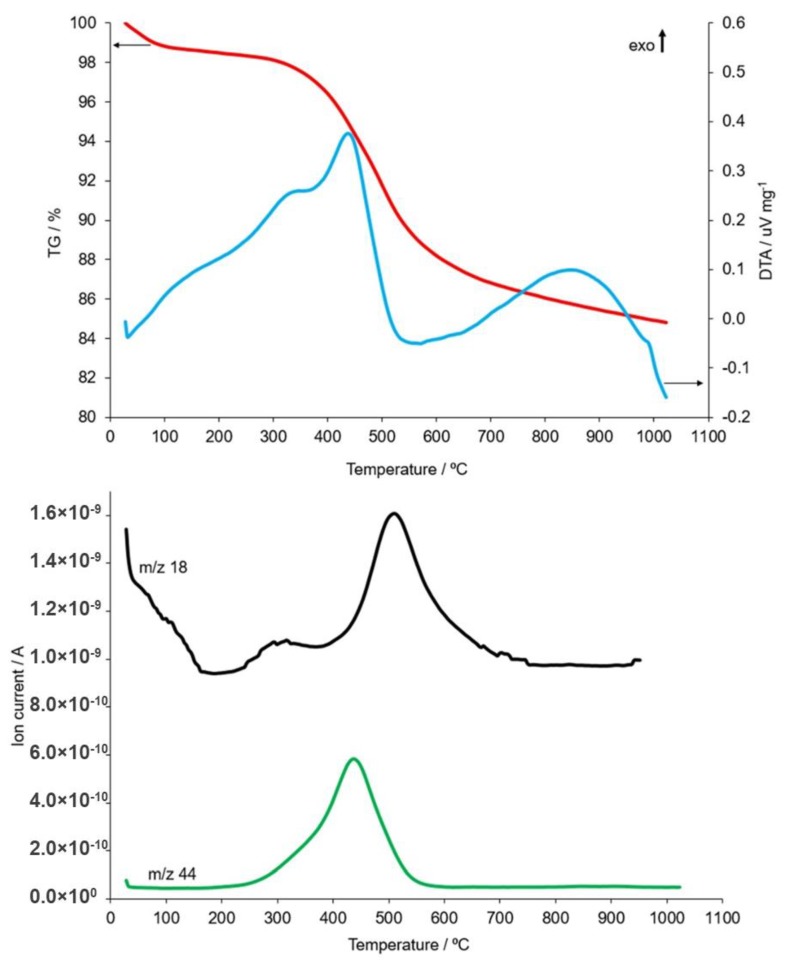
TG, DTA and MS curves (changes in content: H_2_O—*m/z* 18 and CO_2_—*m/z* 44) recorded during heating from ambient temperature up to 1000 °C of the R1 sample taken from the Piast hard coal mines.

**Figure 2:**
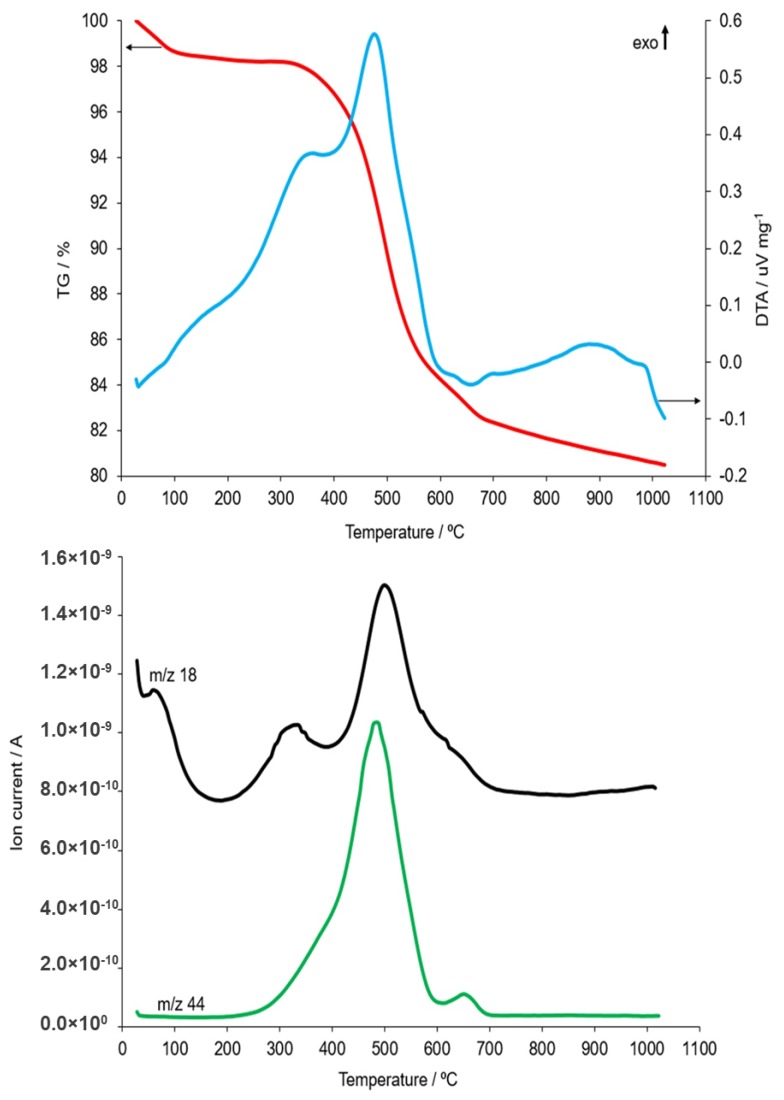
TG, DTA and MS curves (changes in content: H_2_O—*m/z* 18 and CO_2_—*m/z* 44) recorded during heating from ambient temperature up to 1000 °C of R2 sample taken from the *Ruch Rydułtowy* hard coal mines.

Usually, the process of calcination of raw materials is carried out in the temperature range from 550 °C to 700 °C. However, as the research shows, the use of a higher temperature and a longer period of time allows for a higher degree of dehydroxylation [19]. In order to determine the optimal transformation temperature of kaolin present in the studied coal shales, metakaolinite measurements were taken by means of the coupled thermal analysis method. The obtained results are presented in Figure 1 and Figure 2, respectively, from a sample taken from the Piast and Ruch Rydułtowy hard coal mines.

**Figure 3:**
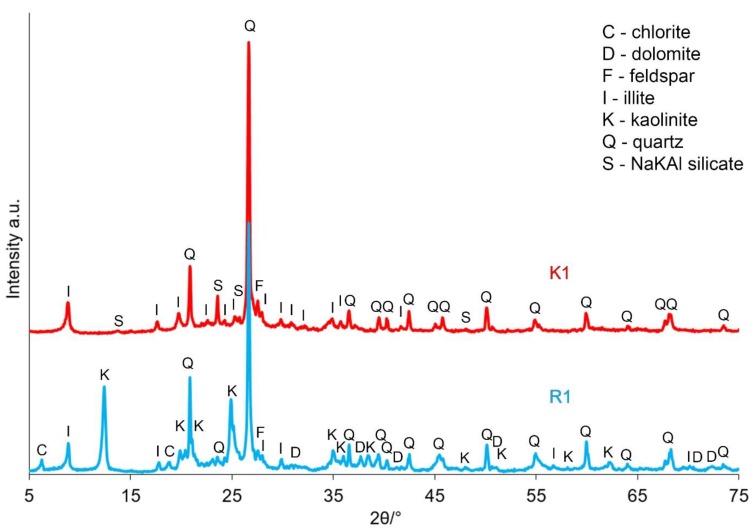
XRD patterns of a sample taken from the Piast hard coal mine, both before (R1) and after the calcination process (K1).

Thermal analysis of the R1 sample showed that the processing of sample components distribution consisted of a multi-stage process. At approximately 225 °C, a dehydration process took place, during which the sample lost 1.6% of its mass. This effect was related to the presence of water that had been adsorbed on the surface of the sample, as well as trapped in the structure of the hydrated salts, e.g., gypsum. The first stage of dehydroxylation was noted within the temperature range from 225 °C to 375 °C. This was evidenced by the increased release of water vapour content (Figure 3), e.g., from goethite type iron hydroxides. In addition, in the temperature range of approximately 180 °C to 600 °C, the decomposition of organic substances in the sample was observed. This effect occurred in two stages, with the exothermic maxima recorded on the DTA curve at 340 °C and 435 °C, respectively (Figure 1). The correctness of such an interpretation of results is confirmed by the extreme change in concentration of CO_2_—*m/z* 44 released from the sample (Figure 1), also recorded at 435 °C. In the temperature range from 380 °C to approximately 790 °C, an intense loss of sample mass occurred, which was associated with a strong endotherm effect recorded on the DTA curve. This was the second stage of the sample’s dehydroxylation process associated with the thermal dissociation of kaolinite and chlorite. This interpretation was additionally confirmed by the increase in intensity emitted from sample at *m/z* 18 (H_2_O). The maximum effect of kaolinite dehydroxylation, which was present in a sample to a much greater extent than chlorite (Figure 3), occurred at 515 °C. A lack of the endothermic effect on this transformation on the DTA curve was related to the occurrence of a much stronger exothermic effect, which occurred within the same temperature range and was associated with the oxidation of organic substances. Chlorite decomposition occurred in the 700–730 °C temperature range (Figure 1). This was evidenced by a slight increase in the water vapour content of the gas of the sample decomposition and a very weak endothermic effect from the extreme value at a temperature of 725 °C. In addition, the conversion of low-temperature β-quartz into the high-temperature variant α-quartz occurred at 573 °C, as evidenced by the endotherm effect recorded on the DTA curve. Subsequent heating of the sample resulted in the formation of metakaolinite as a thermal kaolinite decomposition. The process started at a temperature of approximately 575 °C and the maximum exothermic effect was recorded at a temperature of approximately 845 °C. The transformation was completed at 980 °C. The illite decomposition process began at temperatures above 945 °C. The maximum exothermic effect associated with the destruction of this phase occurred at a temperature of 990 °C.

**Figure 4:**
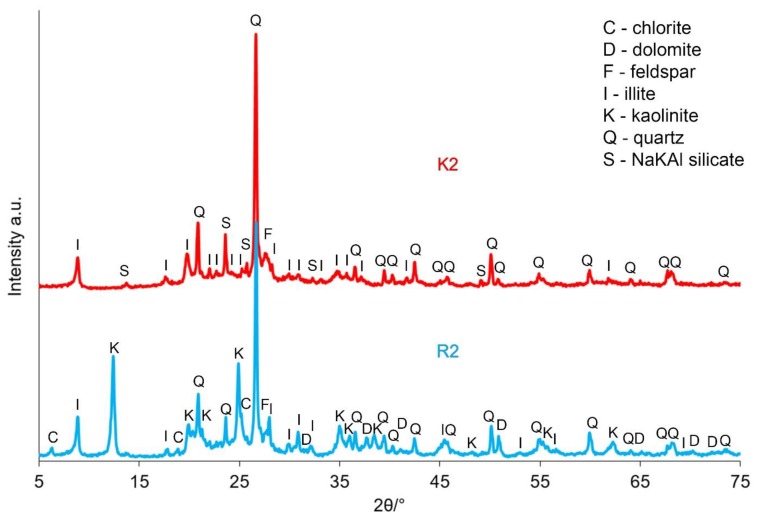
XRD patterns of the sample taken from the Ruch Rydułtowy hard coal mine, both before (R2) and after the calcination process (K2).

Thermal analysis of the R2 sample also showed a multi-staged process of the distribution of its components, similar in nature to the effects recorded for the R1 sample. Dehydration occurred up to an approximate temperature of 200 °C, during which, two components could be distinguished. The first of these was related to the release of moisture adsorbed on the sample’s surface. The extreme endothermic effect for this moisture release was recorded at 80 °C. The second component was related to the distribution of hydrated salts, e.g., gypsum. The extreme value of the very weakly visible endothermic effect was recorded at a temperature of 190 °C. The first stage of the dehydroxylation process took place within the temperature range of 220 °C to 400 °C. This was evidenced by the increase in the water vapour content (*m/z* 18) present in the gas produced from the sample distribution (Figure 2). This effect is characteristic of goethite iron hydroxides. No peaks derived from goethite were detected on the XRD patterns of the unroasted sample (Figure 4), meaning that its content in the sample was small. The decomposition of organic substances in the sample was observed in a wide temperature range from about 220 °C to 600 °C. This phenomenon was a two-stage process with maxima at 355 °C and 475 °C. This was confirmed by exothermic effects with maximum values at these temperatures and changes in CO_2_ concentration (*m/z* 44) released from the sample (Figure 4). A second dehydroxylation step of the sample followed the thermal dissociation of kaolinite and chlorite within the temperature range from 395 °C to about 780 °C. This was evidenced by changes in the water vapour content of the gas produced from the sample decomposition. The maximum effect of kaolinite dehydroxylation, which was present in the sample to a much greater extent than chlorite (Figure 3), occurred at 505 °C. This was confirmed by the maximum concentration of water vapour released in the form of a gaseous decomposition recorded at the mentioned temperature. It was difficult to notice the maximum endotherm effect for this transformation, as it overlapped with a strong exothermic effect related to the oxidation of organic substances. The effects of thermal dissociation of carbonates, most likely of the siderite, were observed in the temperature range of 610–700 °C. This was confirmed by the increase in the intensity of the CO_2_ (*m/z* 44) released from the gas sample products in the dissociation process. In addition, the extreme value of the endothermic effect was recorded at 650 °C. At the temperature of approximately 670 °C, the beginning of the metakaolinite synthesis process, a new phase that arises from the kaolinite thermal decomposition products, was noted. The maximum exothermic effect of this process occurred at a temperature of about 880 °C. The synthesis ended at 980 °C. It was found that the K1 sample produced more of this phase than in the K2 sample when comparing the intensities of the exothermic effect recorded during the heating of R1 and R2 samples, which was observed at temperatures above 575 °C and demonstrated the occurring metakaolinite synthesis processes. In addition, for R1, this process occurred at a temperature that was about 100 °C lower than that of R2. The observed differences were related to the different chemical composition of the tested coal shale. Additionally, sample R1 probably contained more kaolinite and as a result, the effect of its exothermic transformation into amorphous metakaolinite was more intense. The process of the final illite distribution began at temperatures above 975 °C. The maximum exothermic effect associated with the destruction of the structure occurred at a temperature of 985 °C. 

Based on the obtained test results, it was found that the optimal temperature at which the calcination process of materials R1 and R2 should be carried out was at a temperature of 750 °C. This was the lowest temperature at which the kaolinite dehydroxylation process can be completed, which allows for obtaining metakaolinite. 

Figure 3 and Figure 4 present the results of the XRD analysis of the tested samples, both before and after the calcination process, depending on the sampling point. 

Based on the registered diffraction spectra, it was found that in the samples’ mineral composition, the dominating phases were quartz, kaolinite and illite, regardless of the sampling point. These materials also included dolomite, chlorite and feldspar. The calcination process allowed for transforming kaolinite into metakaolinite, which was required for the zeolite synthesis processes. After the calcining process was applied at a temperature of 750 °C, the presence of metakaolinite, directly resulting from the kaolinite phase transition, was observed in both the K1 and K2 samples. Additionally, dolomite and chlorite were not found. 

**Figure 5:**
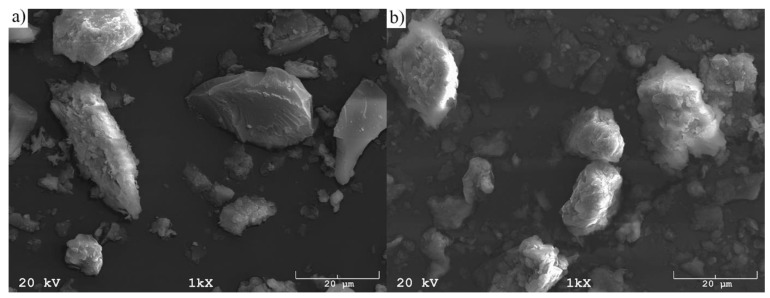
The SEM morphology of coal, depending on the sampling point: (**a**) R1 and (**b**) R2.

**Figure 6:**
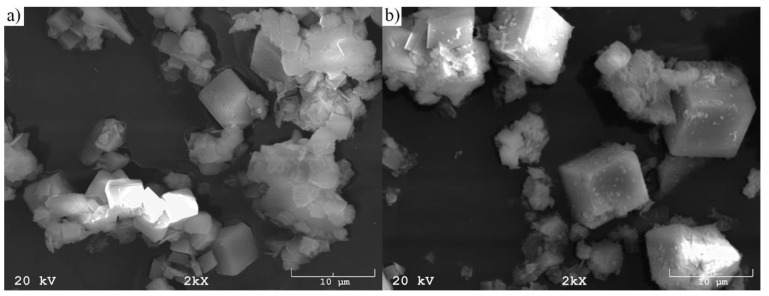
The SEM morphology of produced coal shale synthesis: (**a**) S1 and (**b**) S2.

Figure 5 shows representative pictures of the morphology of the R1 and R2 raw materials. Furthermore, Figure 6 presents representative pictures of the morphology of produced synthesis particles (S1 and S2). 

It was observed that the shape of the raw materials, irrespective of sampling point, was irregular, usually spongy, although some particle surfaces showed shattered surfaces. On the other hand, all the pictures presenting the produced synthesis showed regular cubic solids, being that of zeolite crystals. Their shape was very similar regardless of the raw material’s sampling point. However, the average size of the resulting crystals was almost half the size (about 5 micrometers) compared to when the material extracted from the Piast hard coal mine was used as the raw material. 

The results of the tests conducted on the water solubility of elements for calcination and synthesis samples are presented in Table 3. The obtained results confirmed the safety of using the products acquired as a result of the synthesis of coal shale zeolites. The high content of some elements and compounds, including Ca and SO_4_, among others, in the raw material were rinsed out of the material as a result of the synthesis processes and removed along with the post-process solution. The pH of the obtained materials did not change significantly in relation to the parameters measured in the raw materials collected for testing.

Table 4 presents the results of the chemical composition analysis of the materials obtained after synthesis. In relation to the oxide composition of calcined carbide shales (Table 2), a higher content of Na_2_O was observed. This effect was the result of conducting the synthesis process in a NaOH solution. In addition, there was also a higher content of CaO, which was related to the decomposition of calcium carbonates in the calcination processes.

**Figure 7:**
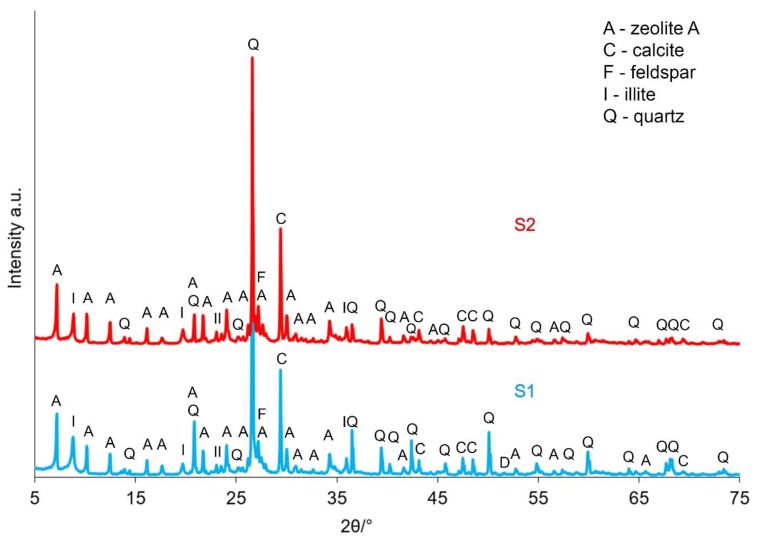
XRD patterns of samples depending on the sampling point: S1 and S2.

Figure 7 presents the results of X-ray analysis of the obtained materials after synthesis depending on the sampling point. 

Based on the conducted Rietveld analysis for the K2 waste (from the Ruch Rydułtowy coal mine) it was confirmed that the kaolin phase was about 22% in the studied material. After the calcination process, it should be assumed that all kaolinite had transformed into metakaolinite. The diffractometric analysis could not determine amorphous substances, such as metakaolinite. However, with the following series: kaolinite-metakaolinit-spinel-mullit-krysobalit, one can assume that if there was no spinel phase, mullite or cristobalite, the whole kaolinite was transformed into metakaolinite. In the conducted synthesis processes under the conditions that have been adopted, all metakaolinite was synthesised into zeolite phases. It can, therefore, be assumed that about 22% of zeolites were obtained (Figure 7). This result was obtained irrespective of the type of raw material used. In the structure of sorbent materials, the occurrence of illite, quartz, calcite and potassium feldspar was also noted (Figure 7). The presence of these minerals indicates incomplete reactivity of substrates during the ongoing synthesis at a temperature of 80 °C for a period of 24 h. It is widely known that raw materials subjected to synthesis are never able to be completely transformed into zeolite phases, which directly reduces the efficiency of the materials produced in this manner. Unreacted phases are referred to as being unreactive [6].

Table 5 presents the results of an analysis of the specific surface and porosity of materials after the calcination process, as well as after the synthesis, depending on the sampling point.

The analysis of the results obtained confirmed that the applied modification to the hydrothermal conditions allowed not only for a change in the phase composition, but also the micromorphology of materials. It was found that after synthesis, the dominant type of porosity in samples was that of micropores. Their share in relation to the total volume of the measured porosity ranged from 53 to 65%. On the other hand, the share of macropores in these materials was small and did not exceed 8%. The remainder were mesopores. The largest measured S_BET_ surface area values were also recorded for these materials, increasing for sample S2 to 172 m^2^/g, and 140 m^2^/g for sample S1. The specific surface values obtained after the synthesis process were over fourteen and seven times higher than those measured for the same materials after the calcination process.

On the basis of a literature research, it can be clearly indicated that zeolite A obtained from synthesis—marked in Table 6 as ML, Na-A, ZF and Syn 4A—has always had a smaller specific surface area than commercial zeolite A, whose specific surface area is contained in the range from 405 to 559.1 m^2^/g. Moreover, Seliem et al. showed that zeolites with similar specific surface area results to the values obtained for sample S2 can be successfully used to remove copper impurities from aqueous solutions. Additionally, the A-type zeolite is one of the most common synthetic zeolites, which has various applications in industry, such as for catalysis, separation, ion exchange or adsorption [45].

## 4. Conclusions

The conducted research confirmed the possibility of managing coal shale coming from a hard coal mine in order to obtain synthetic zeolite materials. Measurements of the thermal analysis allowed for not only characterisation of the phenomena occurring during the heating of materials but enabled the selection of the calcination temperature of raw materials, which was to 750 °C. In addition, based on the recorded exothermic effects, it was found that the R1 material had a higher metakaolin content after synthesis. However, further research has shown that higher metakaolin content does not contribute to better effects regarding sorbent materials’ synthesis. Regardless of the sampling point of the coal shale for testing, zeolite A was always obtained after the synthesis process. The largest specific surface area of 172 m^2^/g was measured on a sample from the Ruch Rydułtowy hard coal mine.

## Figures and Tables

**Table 1:** Description of samples depending on the sampling point and the treatment applied.

Sampling Point	Description of Samples Depending on the Method of Their Preparation		
	Raw Material	After the Calcination Process	After the Synthesis Process
The Piast coal mine	R1	K1	S1
The Ruch Rydułtowy coal mine	R2	K2	S2

**Table 2:** Chemical composition of coal gangue.

Oxides (%)	Description of Samples	
	K1	K2
TiO_2_	1.11	0.88
SiO_2_	65.77	56.84
Al_2_O_3_	21.44	27.37
Fe_2_O_3_	4.22	4.96
CaO	0.33	1.31
MgO	1.26	1.80
Na_2_O	0.73	1.21
K_2_O	3.93	3.67
LOI	0.59	1.17

**Table 3:** The water leaching of ions and the pH of the samples after the calcination process: K1 and K2, and their synthesis: S1 and S2.

Ions	K1 (mg/dm^3^)	K2 (mg/dm^3^)	S1 (mg/dm^3^)	S2 (mg/dm^3^)
Al^3+^	0.611	0.045	2.648	6.051
As	<0.001	0.086	<0.001	0.002
Ca^2+^	11.66	86.84	1.47	1.26
Cd	0.002	0.002	0.003	0.002
Cl	4.54	8.43	5.45	3.58
Co	0.004	0.001	0.005	0.003
Cr	0.011	0.368	0.005	0.001
Cu	0.002	0.002	0.008	0.010
Fe^3+^	0.154	<0.001	1.897	1.420
Hg	0.004	<0.001	<0.001	<0.001
HCO_3_	1.023	3.456	1.112	1.349
K+	6.11	12.42	2.52	2.83
Li	0.076	0.101	0.100	0.075
Mg^2+^	5.92	2.97	1.331	1.703
Na^+^	8.29	27.96	57.05	59.85
Ni	0.004	<0.001	0.010	0.004
PO_4_^3−^	1.70	0.803	0.722	1.370
Pb	<0.001	<0.001	<0.001	<0.001
SO_4_^2−^	189.90	710.19	10.01	30.74
Se	0.294	0.163	0.377	0.417
Si	11.87	38.46	18.11	39.41
Ti	0.118	<0.001	0.979	0.335
pH	6.80	8.65	8.30	8.50

**Table 4:** Results of the chemical composition analysis of samples after synthesis.

Oxides (%)	Description of Samples	
	S1	S2
TiO_2_	0.85	0.68
SiO_2_	51.12	41.78
Al_2_O_3_	17.19	21.31
Fe_2_O_3_	3.37	3.68
CaO	9.73	10.53
MgO	1.15	1.75
MnO	0.03	0.06
SrO	0.04	0.04
BaO	0.06	0.05
Na_2_O	2.08	2.95
K_2_O	3.02	2.47
P_2_O_5_	0.16	0.09
SO_3_	0.08	0.13
ZrO_2_	0.07	0.04
LOI	10.94	14.31

**Table 5:** The results of an analysis of the specific surface and porosity of materials after the calcination process and after synthesis, depending on the sampling point.

Description of Samples	S_BET_ (m^2^/g)	$V_{\mathrm{tot}}^{0.99}\;({\mathrm{cm}}^{3}/g)$	$V_{\mathrm{mik}}^{\mathrm{DR}}\;({\mathrm{cm}}^{3}/g)$	$V_{\mathrm{mez}}^{\mathrm{BJH}}\;({\mathrm{cm}}^{3}/g)$	$V_{\mathrm{mak}}\;({\mathrm{cm}}^{3}/g)$
K1	18	0.031	0.005	0.019	0.007
S1	140	0.083	0.053	0.026	0.004
K2	12	0.035	0.005	0.020	0.010
S2	172	0.096	0.065	0.019	0.008

S_BET_ (m^2^/g)—specific surface according to Brunauer–Emmett–Teller (BET) theory; $V_{\mathrm{tot}}^{0.99}$ (cm^3^/g)—total specific volume of pores for a relative pressure p/p_0_ = 0.99; $V_{\mathrm{mik}}^{\mathrm{DR}}$ (cm^3^/g)—the volume of micropores (pores with widths under 2 nm) according to the Dubinin–Radushkevich method; $V_{\mathrm{mez}}^{\mathrm{BJH}}$ (cm^3^/g)—the volume of mesopores (pores with a width greater than 2 nm and less than 50 nm) according to the Barrett–Joyner–Halve (BJH) method; $V_{\mathrm{mak}}$ (cm^3^/g)—the volume of macropores (pores wider than 50 nm).

**Table 6:** The specific surface area of obtained materials and synthetic zeolites of type A.

Description of Samples	S_BET_ (m^2^/g)	References
S1	140	in article
S2	172	in article
ML	328	[46]
Na-A	39	[47]
ZF	189	[48]
Comm 4A	559	[49]
Syn 4A	22	[49]
Commercial 3A	450	[50]
Commercial 4A	405	[50]
